# Utility of citizen science data: A case study in land-based shark fishing

**DOI:** 10.1371/journal.pone.0226782

**Published:** 2019-12-19

**Authors:** Kesley J. Gibson, Matthew K. Streich, Tara S. Topping, Gregory W. Stunz

**Affiliations:** Harte Research Institute for Gulf of Mexico Studies, Texas A&M University–Corpus Christi, Corpus Christi, Texas, United States of America; Australian Bureau of Agricultural and Resource Economics and Sciences, AUSTRALIA

## Abstract

Involving citizen scientists in research has become increasingly popular in natural resource management and allows for an increased research effort at low cost, distribution of scientific information to relevant audiences, and meaningful public engagement. Scientists engaging fishing tournament participants as citizen scientists represent ideal scenarios for testing citizen science initiatives. For example, the Texas Shark Rodeo has begun shifting to conservation-oriented catch-and-release practices, which provides a unique opportunity to collect data on a large scale for extended periods of time, particularly through tagging large numbers of sharks for very little cost compared to a directed scientific study. However, critics are somewhat skeptical of citizen science due to the potential for lack of rigor in data collection and validation. A major management concern for shark fisheries is the ability of anglers to identify species. We tested some of the assumptions and value of citizen-collected data by cross-verifying species identification. Specifically, the purpose of this study was to evaluate the accuracy of shark species identifications made by anglers fishing in the Texas Shark Rodeo using photographs that were submitted as a requirement for tournament participation. Using a confusion matrix, we determined that anglers correctly identified 97.2% of all shark catches submitted during the Texas Shark Rodeo from 2014–2018; however, smaller sharks and certain species, including blacknose and spinner sharks, were more difficult to identify than others. Most commonly confused with blacktip sharks, spinner sharks were most commonly identified incorrectly (76.1% true positive rate [TPR]) followed by blacknose (86.8% TPR), finetooth (88.0% TPR), and Atlantic sharpnose sharks (93.8% TPR). This study demonstrated that citizen scientists have the ability to identify sharks with relatively low error. This is important for science and management, as these long-term datasets with relatively wide geographic scope could potentially be incorporated into future assessments of sharks in the Gulf of Mexico.

## Introduction

While not a new concept, the use of citizen scientists, or volunteers to aid in the collection of data as part of scientific inquiry, has become increasingly popular in ecology and natural resource management. Historically, citizen scientists have been a privileged few, including names such as Benjamin Franklin and Charles Darwin, working either alone or with other amateurs [1]. Today, the availability of technology and encouragement from the scientists and granting agencies seeing value in stakeholder engagement and outreach has helped increase dissemination of information to and ultimately provide more meaningful involvement by citizen scientists in a wide range of fields. Citizen scientists’ interest in scientific investigations stem from a variety of factors, but may include a study’s geographic location (e.g., favorite fishing or hunting area), particular focal species or group (e.g., birds, fish), or general topic (e.g., climate change). This has allowed for beneficial relationships to form between the scientific community and the public, allowing researchers additional personnel to collect samples and promoting more meaningful public engagement typically through hands-on learning experiences [2].

Resource managers have worked with citizen scientists on a diverse array of research topics including ornithology, reef ecology, community composition, and water quality monitoring among others [3–8]. One of the most iconic and longest on-going examples of citizen science is the Christmas Bird Count run by the National Audubon Society in the United States every year since 1900, which involves thousands of amateur birders who help to perform surveys that would be impossible for just a few scientists to efficiently complete [9]. These participants are a large workforce that have already contributed significant scientific knowledge regarding range expansions and distribution patterns of North American birds [10]. Within the field of fisheries, managers have recently begun using mobile apps to gather fisheries-dependent data from recreational anglers [11–12]. Engaging recreational anglers has provided data from remote locations that might have otherwise been logistically or financially inaccessible [13]. Thus, there are clear opportunities to advance science and improve the spatial and temporal scope of studies by involving citizen scientists.

Despite these gains in research capacity, there are some challenges with citizen science, including standardizing the collection method and maintaining scientific rigor and validity of the data [1–2,10,13–14]. These challenges contribute to the scientific community’s uncertainty about the reliability of data collected by citizen scientists [5,15]. Foster-Smith and Evans [10] explored the reliability of volunteer-collected data by comparing it to data collected by professionally trained scientists. Volunteers assisted scientists in mapping the distribution and abundance of common coastal species along the shores of Isle of Cumbrae, Scotland. The study found that volunteers were capable of learning to identify species, record their occurrence, and take size measurements. While some errors by volunteers were detected during the study, similar errors were also detected from the trained scientists, including recording errors and some species misidentification, but that accuracy improved with practice. Misidentification errors can have serious impacts on ecological monitoring studies and conservation actions, including culling of endangered species [16], unobserved declines in fish stocks [17], and wasted resources through drafting of inappropriate management plans for species with false sightings [18]. Nevertheless, these studies showed there is real potential for citizens to add valuable and credible data to the scientific knowledge base.

**Fig 1:**
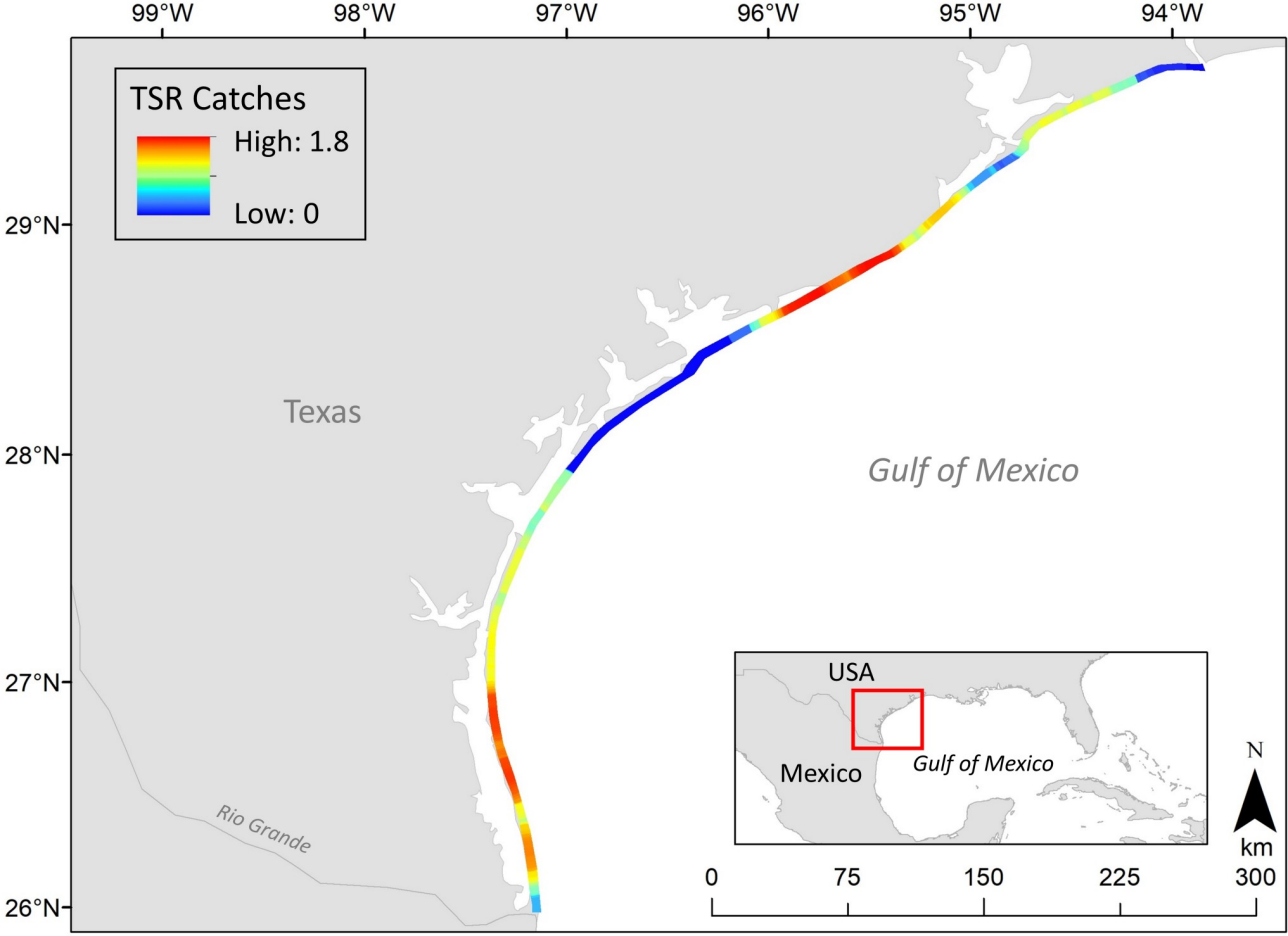
Map of Texas coastline showing hotpots of land-based sharks catches during the Texas Shark Rodeo, 2014–2018. Shark catches are depicted using point density (number of sharks/km^2^) over a grid of 50 x 50 m cells. Base-maps are used freely from Natural Earth (naturalearthdata.com).

We had the opportunity to explore and validate the value of scientific data for shark fisheries along the Texas Coast. The Texas Shark Rodeo (TSR) is an annual 9-month long land-based shark fishing tournament that advocates for catch-photo-release with an “emphasis on tagging and collecting data for the conservation of sharks” (texassharkrodeo.com; Fig 1). There is no entry fee for the tournament, with winners receiving trophies and recognition, but no monetary incentive. Anglers participating in the TSR tag and submit a photograph of their catch for it to be counted. This allowed for validation at several levels: the angler, tournament official, and scientist. Interestingly, the tagging component also allowed for a second verification by anglers if a shark was recaptured. At the end of the tournament, the participant or team with the most points wins, depending on the division (e.g., top three teams, top three anglers, top three junior anglers, and largest of each species). Participants earn points based on the length of sharks landed with the potential to earn bonus points for collecting scientific data, landing an “uncommon” species, or recapturing a previously tagged shark. While species misidentification is a valid concern in many citizen science efforts [19–21], access to inexpensive cameras and equipment, many of which are integrated into smartphones, afford scientists the ability to verify observations made by citizen scientists [22]. The purpose of this study was to evaluate the accuracy of shark species identifications using submitted photographs to determine the validity of this citizen science-generated data for use in scientific investigation.

## Methods

Anglers participating in the TSR were permitted to target sharks from shore (e.g., beach, jetty, channel), excluding piers or vessels of any type. As per tournament rules, landed sharks were identified, measured, photographed, tagged with a conventional dart tag (if length was ≥32 in [81.3 cm]; tournament rule), and released. Date of capture, location, stretched total length (STL; measured from the tip of the snout to the tip of the stretched upper caudal lobe), sex, species, and tag number, along with photographs were then submitted via online form. These data were available for all sharks captured during the TSR between 2014 (first year of the tournament) and August 2018.

To confirm the species identification submitted by anglers, each photograph was viewed during the data entry and quality control process by tournament officials and then by expert scientists (first reader). If the angler species identification was not confirmed by the first reader, the photograph was sent to the second reader for a blind identification. If identifications differed between the two readers, then a third reader made a blind identification of the photograph. If identifications still differed, then the photograph was jointly examined by the second and third readers, and if a consensus could not be reached, the shark was classified as unknown. Percent agreement was calculated between the anglers and the final reader identification excluding those classified as unknown.

**Fig 2:**
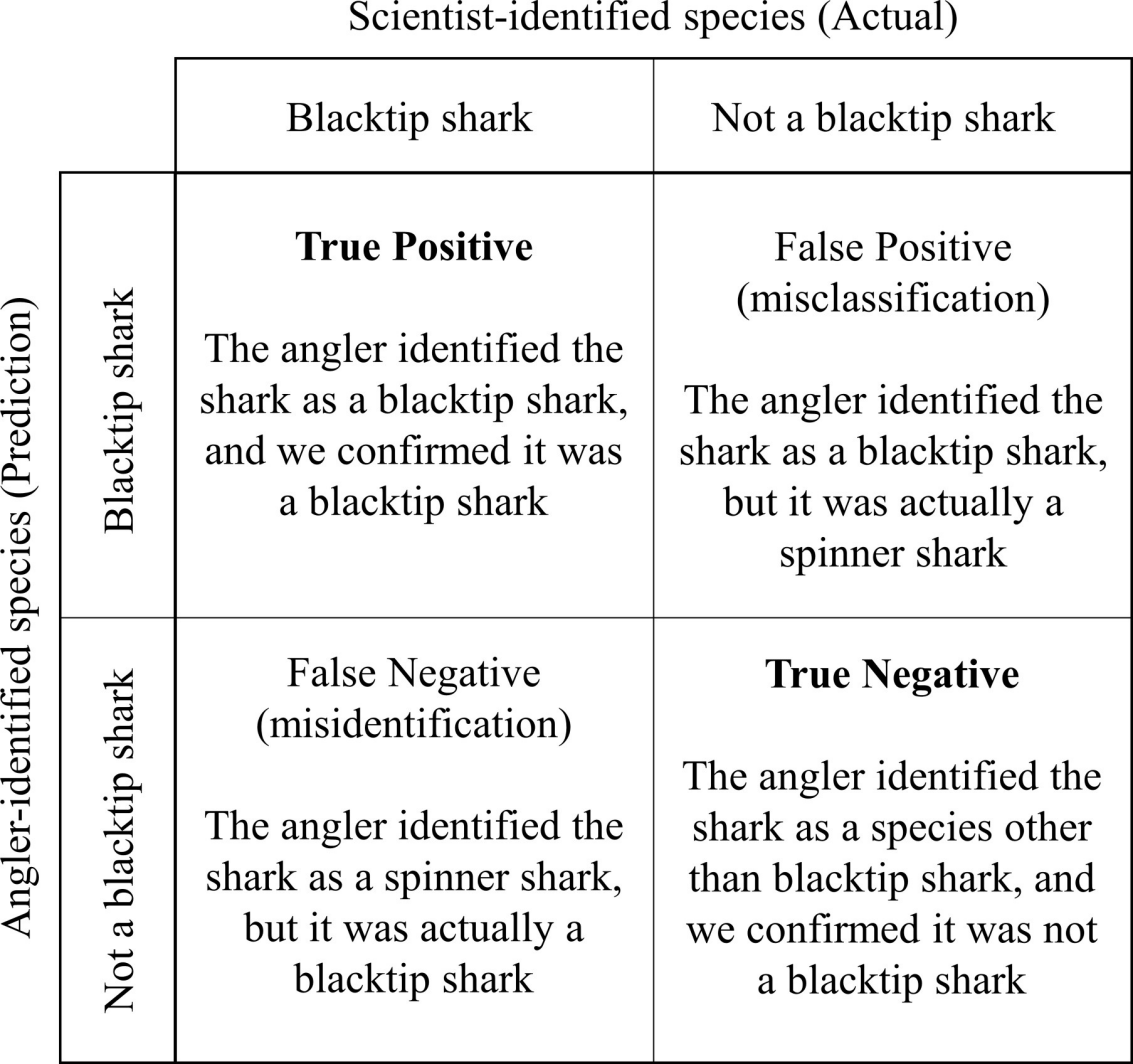
Example confusion matrix for binary classification of blacktip sharks. An example for each correct (true positive; true negative) and incorrect (false negative; false positive) outcome is given within each cell.

Statistical analyses were completed in *R* version 3.5.0[23] to determine accuracy of shark species identification and for reader comparison analyses. Confusion matrices using the *caret* package [24–25] were used to determine the overall accuracy of angler identification of shark species for the entire dataset and by size binned into 25-cm increments. Prediction and classification error were also determined for the entire dataset and by size. For the purpose of this study, species identifications made by anglers were considered predictions and were compared to the species identifications made by the scientists, which were considered the validated or actual species. Therefore, prediction errors (also known as false negatives or misidentifications) occurred when the angler identified the shark as a species other than the species identified by skilled scientists (e.g., the angler identified the shark as a spinner shark (*Carcharhinus brevipinna*), but it was actually a blacktip shark [*Carcharhinus limbatus*]). Conversely, classification errors (also known as false positives or misclassifications) occurred when the angler identified the shark as one species when it was in fact another species (e.g., the angler identified the shark as a blacktip shark, but it was actually a spinner shark; Fig 2). Using a one-sided exact binomial test, the overall accuracy rate was compared to the no-information rate (NIR), the best ‘guess’ of species if no information was given which corresponds to the largest proportion of the observed classes. If the overall accuracy was significantly greater than the NIR, angler species identifications were significantly better than identifications based on chance [24]. McNemar’s test was used to assess the symmetry of the species identification agreement table (i.e., angler vs. scientist identifications). A significant test was expected if certain species were consistently misidentified. This analysis was followed up with one-sided exact binomial tests to determine if the probability of anglers correctly identifying certain shark species was lower than the overall accuracy rate. All tests were conducted at the *α* = 0.05 significance level.

### Ethics statement

This research was approved by the Texas A&M University-Corpus Christi Institutional Animal Care and Use Committee under protocols #08–15 and #08–18 and also by National Park Service permits PAIS-2010-SCI-0009, PAIS-2015-SCI-0001, and PAIS-2016-SCI-0018. Tournament participants captured and tagged sharks following the rules and regulations of the Texas Parks and Wildlife Department and the TSR. The individuals in this manuscript have given written informed consent (as outlined in PLOS consent form) to publish these case details.

## Results

Participants of the TSR submitted 5,419 unique sharks with corresponding data and photographs from 2014 through August 2018. Of those submissions, only one submission did not include a photograph of a shark, leaving 5,418 unique sharks in the data set. Blacktip sharks were the most frequently captured species followed by bull sharks (*Carcharhinus leucas*) then sandbar sharks (*Carcharhinus plumbeus*; Table 1). The largest shark captured was a tiger shark (*Galeocerdo cuvier*) at 427 cm STL, and the smallest was a sandbar shark measuring 33 cm STL. At least one shark was reported in each size bin covered by that species possible biological size range.

**Table 1:** List of shark species caught during the Texas Shark Rodeo (TSR) from 2014 to August 2018 in order from most frequently captured to least frequently captured, including minimum, maximum and average stretch total length (STL; cm) for each species. Recaptures were also reported for sharks tagged and recaptured by participants in TSR (recaptures during TSR) and sharks tagged during TSR but recaptured by a non-participant of TSR (recaptures outside of TSR).

Species	Number Caught	Percent Total	Min STL (cm)	Max STL (cm)	Average STL (cm)	Recaptures during TSR	Recaptures outside TSR
Blacktip	2526	46.6	43	200	143	18	22
Bull	1581	29.2	74	284	173	12	6
Sandbar	354	6.5	18	267	133	15	6
Atlantic sharpnose	224	4.1	33	135	83		
Spinner*	163	3.0	60	231	103	1	2
Bonnethead	144	2.7	46	117	70		1
Finetooth*	117	2.2	48	152	109		
Blacknose*	68	1.3	46	133	105		1
Scalloped hammerhead*	68	1.3	43	262	167		
Great hammerhead*	50	0.9	188	396	283		2
Tiger*	50	0.9	122	427	252		1
Lemon*	41	0.8	130	295	236	4	1
Unknown	28	0.5	43	99	68		
Dusky*	2	<0.1	290	293	291		
Shortfin mako*	2	<0.1	320	330	325		
**Total**	**5418**	**100**	**18**	**427**	**147**	**50**	**42**

*uncommon species designation under TSR rules.

A total of 92 recaptures were reported during the study period for a 1.7% recapture rate; however, eight of the recaptured sharks were originally tagged outside of the TSR (e.g., four were tagged as part of the National Marine Fisheries Service (NMFS) Apex Predator Program). Of the 92 recaptures, 42 were reported as by tournament anglers (i.e., sharks tagged and recaptured by participants in the TSR), and 42 recaptures were reported outside of the TSR (i.e., sharks tagged during the tournament but recaptured by a non-participant of the TSR). Of the 42 recaptures reported as part of the TSR, seven sharks were harvested or died prior to release, and two were reported dead after washing up on the beach shortly after the initial tagging event. Of the 42 recaptures reported outside of the TSR, seven sharks were reported washed up on the beach after the initial tagging event. Not including death during the fight or landing process, 9.8% (n = 9) of recaptures were reported as washed up or dead upon recapture. Two sharks were recaptured twice, a sandbar shark and a great hammerhead shark (*Sphyrna mokarran*).

**Fig 3:**
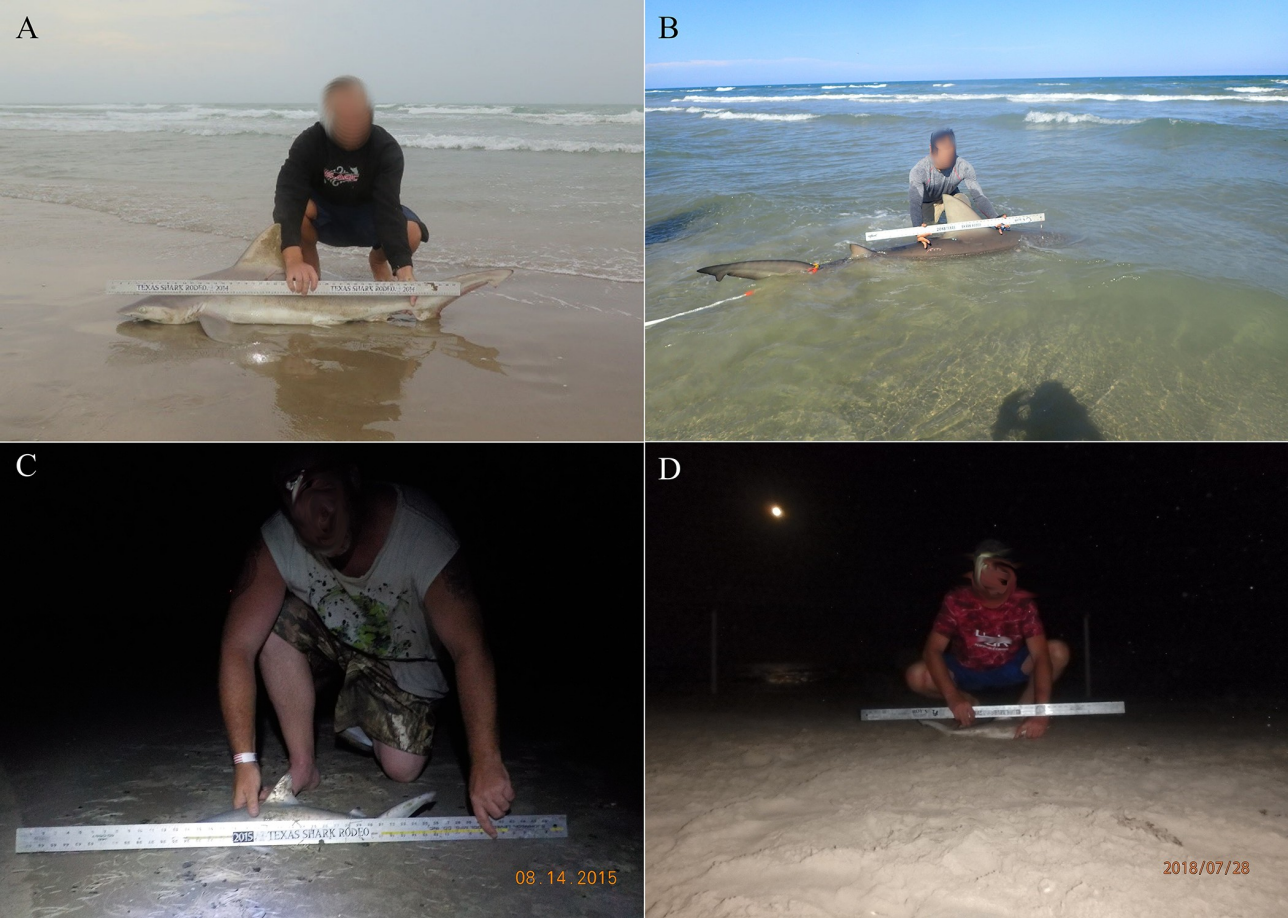
Shark photographs submitted by participants in the TSR where distinguishing characteristic were visible (A) and photographs that were classified as unknown (B-D). A) Confirmed sandbar shark with visible characteristics. B) Angler identified shark as bull shark, but identification could not be confirmed because shark was underwater. C) Angler identified shark as blacktip shark, but identification could not be confirmed because ruler covered most of the shark’s body. D) Angler identified shark as a sandbar shark, but species could not be identified because of photo scale, blurriness, and shark’s head covered by angler’s hand.

During visual confirmation of angler species identification, readers classified 28 entries (0.5% of all shark photographs) as unknown. The unknown classification was assigned to shark photographs that could not be identified because distinguishing characteristics were not visible (Fig 3). Readers were able to assign a species identification to the remaining 5,390 photographs. After the final reading, the readers and anglers agreed on 97.9% of the identifications. Overall, 125 sharks were misidentified by anglers (Table 2), which corresponded to a 97.2% (95% CI: 96.7–97.6) overall accuracy in angler identification of sharks that were positively identified by the readers. The NIR was calculated at 46.6%, which was significantly different from the overall accuracy rate (p < 0.001), suggesting that anglers could not just guess the species identification and be correct the majority of the time.

**Table 2:** Shark identification agreement table comparing angler species identifications (rows) to the those made by scientists (columns; considered the actual identification) for sharks captured, photographed, and released during the TSR between 2014–2018. Shaded numbers are true positives (e.g., identifications by anglers and scientists agree). Numbers above the shaded numbers are misclassifications and numbers below true positive identifications are misidentifications.

	Atlantic sharpnose	Blacknose	Blacktip	Bonnethead	Bull	Dusky	Finetooth	Great hammerhead	Lemon	Shortfin mako	Sandbar	Scalloped hammerhead	Spinner	Tiger	Unknown	Misclassified
Atlantic sharpnose	210	1	4	0	0	0	5	0	0	0	0	0	4	0	3	17
Blacknose	0	59	2	0	0	0	0	0	0	0	0	0	0	0	0	2
Blacktip	8	2	2489	0	1	0	6	0	0	0	2	0	31	0	14	64
Bonnethead	0	0	0	143	0	0	0	0	0	0	0	0	0	0	0	0
Bull	0	0	4	0	1579	0	0	0	0	0	5	0	0	0	1	10
Dusky	0	0	0	0	0	2	1	0	0	0	0	0	3	0	0	4
Finetooth	0	6	3	0	1	0	103	0	0	0	0	0	0	0	1	11
Great hammerhead	0	0	0	1	0	0	0	49	0	0	0	1	0	0	0	2
Lemon	0	0	0	0	0	0	0	0	41	0	0	0	0	0	0	0
Shortfin mako	0	0	0	0	0	0	0	0	0	2	0	0	0	0	0	0
Sandbar	3	0	9	0	0	0	1	0	0	0	347	0	0	0	5	18
Scalloped hammerhead	0	0	0	0	0	0	0	1	0	0	0	67	0	0	0	1
Spinner	3	0	15	0	0	0	1	0	0	0	0	0	124	0	4	23
Tiger	0	0	0	0	0	0	0	0	0	0	0	0	0	50	0	0
Unknown	0	0	0	0	0	0	0	0	0	0	0	0	1	0	0	1
**Misidentified**	14	9	37	1	2	0	14	1	0	0	7	1	39	0	28	**153**

Higher error rates were observed for certain species that are more difficult to distinguish. For example, the species identification agreement table failed to pass McNemar’s test of symmetry (χ^2^ = 8.01, df = 1, p = 0.005), suggesting certain species were consistently misidentified by anglers. Exact binomial tests indicated the identification accuracy for four species was significantly less than the overall accuracy rate (p < 0.001). Spinner shark identification accuracy was the lowest (76.1% TPR) followed by blacknose shark (*Carcharhinus acronotus*; 86.8% TPR), finetooth shark (*Carcharhinus isodon*; 88.0% TPR), and Atlantic sharpnose shark (*Rhizoprionodon terraenovae*; 93.8% TPR) (Table 3). Tiger sharks, lemon sharks (*Negaprion brevirostris*), and shortfin mako sharks (*Isurus oxyrinchus*) were the only species with no misidentifications or misclassifications (100% TPR). The dusky shark (*Carcharhinus obscurus*) was also never misidentified (100% TPR) but was misclassified four times as either a spinner or finetooth shark. Some angler-identified species were never misclassified, but were misidentified, such as the bonnethead shark (*Sphyrna tiburo*) which was misidentified once (as a great hammerhead shark) in the 144 encounters (99.3% TPR). The most commonly confused sharks by anglers were blacktip and spinner sharks with 31 of the 39 misidentified spinner sharks initially identified as blacktip sharks. Fifteen of the 37 misidentified blacktip sharks were initially identified as spinner sharks. Excluding shark identifications that could not be confirmed (i.e., unknown), blacktip sharks were misclassified the most with 50 submissions, followed by spinner sharks with 19 misclassifications, and Atlantic sharpnose sharks with 14 misclassifications (Table 2).

**Table 3:** Species sensitivity (TPR) and specificity (true negative rate [TNR]) for all sharks identified by anglers participating in TSR, 2014–2018. Sensitivity (TPR) is also reported for sharks of taggable size in the tournament (81.3 cm STL).

Species	Sensitivity (True Positive Rate)	Specificity (True Negative Rate)	Sensitivity (Taggable size)
Dusky	1.0000	0.9993	1.0000
Lemon	1.0000	1.0000	1.0000
Shortfin mako	1.0000	1.0000	1.0000
Tiger	1.0000	1.0000	1.0000
Bull	0.9987	0.9974	0.9987
Bonnethead	0.9931	1.0000	0.9706
Blacktip	0.9854	0.9779	0.9872
Scalloped hammerhead	0.9853	0.9998	0.9792
Sandbar	0.9802	0.9965	0.9855
Great hammerhead	0.9800	0.9996	0.9800
Atlantic sharpnose	0.9375	0.9967	0.9640
Finetooth	0.8803	0.9979	0.8571
Blacknose	0.8677	0.9996	0.8571
Spinner	0.7607	0.9956	0.8875
Unknown	0.0000	0.9998	0.0000

Overall accuracy of angler identifications for sharks that were eligible for tagging per tournament rules (81.3 cm STL) increased to from 97.2% to 98.4% (95% CI: 98.0, 98.8). Finetooth (85.7% TPR) and blacknose sharks (85.7% TPR) were identified with lowest accuracy, followed by spinner sharks with an 88.8% TPR (Table 3). Blacktip and spinner sharks of these larger size classes remained the most confused species, with 13 blacktip sharks initially identified as spinner sharks and 9 spinner sharks initially identified as blacktip sharks. However, larger blacktip sharks were identified correctly in 98.5% of all encounters. In addition to tiger, lemon, and shortfin mako sharks, dusky sharks were also identified with high accuracy and had no misclassifications at taggable size, but these species comprised a small portion of captured individuals. The number of misidentified sharks decreased from 125 when considering all sharks entered in the TSR to 72 when considering only those ‘eligible’ for tagging. Similarly, sharks with unknown identification decreased from 28 to 2 individuals.

Species identification generally became more accurate as size increased as anglers were 100% accurate for sharks measuring 225 cm STL and larger. However, the data showed finetooth sharks were more difficult to identify as they grew larger, commonly being misidentified as blacktip or Atlantic sharpnose sharks. Blacktip sharks were the most commonly captured species in the 50–175 cm size range and were identified correctly (i.e., TPR) >95% of the time (Table 4). Generally, for all species, smaller sharks were the hardest for anglers to identify, with blacknose sharks between 75–100 cm having the lowest TPR at 50.0%, followed by spinner sharks between 50–75 cm and 100–125 cm at 62.3% TPR and 80.0% TPR, respectively; this was consistent with spinner sharks being the most difficult to identify overall.

**Table 4:** Angler sensitivity (i.e., TPR) by size class for sharks captured during TSR from 2014–2018. Size class was binned into 25-cm increments (e.g., 25 = 0–25 cm). Sample size for each size class by species is represented by the numbers in parenthesis while blank cells mean no sharks were reported for that size class for that species.

Size class (cm)	Atlantic sharpnose	Blacknose	Blacktip	Bonnethead	Bull	Dusky	Finetooth	Great hammerhead	Lemon	Shortfin mako	Sandbar	Scalloped hammerhead	Spinner	Tiger	Unknown
25											1.00				
											(1)				
50	0.83	1.00	1.00	1.00			1.00					1.00	0.00		0.00
	(29)	(3)	(2)	(4)			(1)					(1)	(0)		(0)
75	0.91	0.89	0.95	1.00	1.00	0.00	0.93				0.97	1.00	0.62		0.00
	(46)	(9)	(142)	(102)	(1)	(0)	(27)				(130)	(18)	(60)		(0)
100	0.95	0.50	1.00	0.96	1.00		1.00				0.98	1.00	0.80		0.00
	(101)	(2)	(182)	(25)	(17)		(18)				(45)	(1)	(61)		(0)
125	1.00	0.86	0.97	1.00	1.00		0.88	0.00			1.00		0.71		
	(46)	(49)	(177)	(13)	(43)		(16)	(1)			(5)		(7)		
150	1.00	1.00	0.99		1.00		0.81		1.00		1.00	1.00	1.00		
	(2)	(5)	(777)		(259)		(54)		(2)		(7)	(1)	(1)		
175			0.99		1.00		1.00	1.00	1.00		1.00	1.00	0.80		
			(990)		(578)		(1)	(4)	(3)		(28)	(1)	(5)		
200			0.98		1.00			0.75	1.00		1.00	0.93	1.00	1.00	
			(255)		(425)			(4)	(1)		(47)	(15)	(8)	(1)	
255			1.00		1.00			1.00	1.00		0.99	1.00	0.95	1.00	
			(1)		(156)			(8)	(4)		(81)	(17)	(19)	(1)	
250					1.00			1.00	1.00		1.00	1.00	1.00	1.00	
					(76)			(12)	(15)		(9)	(12)	(1)	(9)	
275					1.00			1.00	1.00		0.00	1.00		1.00	
					(23)			(9)	(10)		(1)	(2)		(12)	
300					1.00	1.00		1.00	1.00					1.00	
					(3)	(2)		(2)	(6)					(12)	
325								1.00		1.00				1.00	
								(4)		(1)				(7)	
350								1.00		1.00				1.00	
								(2)		(1)				(4)	
375								1.00						1.00	
								(1)						(3)	
400								1.00						1.00	
								(1)						(1)	
425								1.00							
								(1)							
450								1.00							
								(1)							

## Discussion

Land-based shark fishing is a popular recreational activity in Texas, with the trend favoring more conservation-oriented practices using catch-and-release methods [3,26–27]. Tournaments that follow these conservation-oriented practices, like the TSR, are dramatically gaining in popularity and participation, and they are also important in providing an alternative to kill-tournaments that can impact stocks, and often face public criticism [28]. The switch to no-kill tournaments and increase in popularity of catch-and-release has provided a unique opportunity to collect data on a large-scale for extended periods of time with minimal costs compared to traditional scientific surveys of this size. The partnership with the TSR has allowed for the documentation of >5,400 sharks by >380 anglers along the entire Texas coast during a ~4-year period–tagging and data collection of a magnitude that would be impossible under traditional scientific study constraints both logistically and financially. Previous studies using species composition and biological measurements collected during the TSR assumed that participants were relatively skilled and adept at shark identification [3,29], and these studies relied on the species identifications being accurate. Given the 97.2% overall accuracy of shark identifications made by anglers in the TSR, our study confirms that this group of citizen scientists can provide reliable data for studies of shark populations off the Texas coast. This accuracy is comparable to other accuracy rates for species identification across a variety of taxa and systems, which have been typically reported between 70–95% [30–32].

While the overall accuracy of shark species identifications was high, some species were more difficult for anglers to recognize. For example, spinner sharks were commonly confused with blacktip sharks. Discerning between these two species has also been an issue with scientists in the past [33]. The key characteristic commonly used to distinguish between these two species in field guides and by anglers is the lack of pigmentation on the anal fin of blacktip sharks that is usually present on spinner sharks greater than 80 cm STL [33]. Smaller spinner sharks (<75 cm STL) were the most misidentified category in this study, likely due to these sub-adult sharks lacking pigmentation on the anal fin, making them visually very similar to blacktip sharks. Overall, spinner sharks were poorly identified in most size classes examined, with anglers tending to classify spinner sharks as blacktip sharks rather than the reverse. Given that spinner sharks were classified as an uncommon species by the TSR and, therefore, earned more points in the tournament than blacktip sharks, it seems most likely that anglers were simply misidentifying them and not intentionally misidentifying them to earn more tournament points. Furthermore, spinner sharks were also misidentified as dusky sharks, Atlantic sharpnose sharks, and one was submitted as unknown. Thus, for species like the spinner shark which are more difficult to identify, it is critical to continue validation of submissions and increase educational opportunities and materials to better prepare anglers when they do encounter these species.

Contrary to similar species such as blacktip and spinner sharks, many sharks have extremely distinguishing characteristics making them easier to identify, especially off the Texas coast. For example, tiger sharks have very characteristic markings (i.e., ‘stripes’) making them distinctive from most species commonly captured in the Gulf of Mexico [34–35]. The shortfin mako is also distinct with its deep blue coloring and dark eye, and while it may be confused with the longfin mako (*Isurus paucus*), the longfin mako is rare off Texas, making this misidentification unlikely. While their hammer-shaped heads make the group iconic, some anglers have confused bonnethead, great hammerhead, and scalloped hammerhead sharks (*Sphyrna lewini*). Adult bonnethead sharks are smaller than adult scalloped or great hammerhead sharks but may be confused with juveniles even though bonnetheads have a more rounded, shovel-shaped head [35]. Great hammerhead and scalloped hammerhead sharks have a more nuanced distinction between the two species. The great hammerhead has a straighter leading edge of the hammer and has a taller dorsal fin than the scalloped hammerhead but could be easily confused if not commonly observed [35]. While all of these species are distinct and more readily identifiable, they comprised only a small portion of the data set (5.8%).

Despite smaller size classes being more difficult to identify for all species, angler identification accuracy was higher than the NIR (i.e., the largest proportion of the observed classes), suggesting that angler identifications were better than identifications made by chance alone. Anglers were unlikely to misidentify sharks in the largest size classes (>275 cm STL), which included less frequently captured species such as dusky, great hammerhead, lemon, shortfin mako, and tiger sharks. As per tournament rules, sharks above 81.3 cm could be tagged, earning the TSR participant additional points. This rule eliminated most of the smaller sharks that were misidentified, especially spinner sharks, which increased to 88.8% TPR when excluding these smaller individuals. Overall identification accuracy of tagged individuals greater than 81.3 cm also increased to 98.4%. These findings clearly indicate size is a factor influencing accurate species identifications. With more training and the development of a field guide for shark pups and early juveniles, anglers may be able to further improve their identification skills.

Recaptures are an important component of any tagging study, as they can provide insight into fish movements, growth rates, catch and survival rates, and site fidelity [36–37]. However, recapture data rely on the angler or beachgoer to report the fish to the proper tagging institution. Of the 92 shark recaptures reported during this study, eight were originally tagged outside of TSR, but were reported during the tournament via the online form. In the tournament, anglers received bonus points for recaptured sharks even if the tag was not distributed by the tournament, giving incentive to report any recapture. Additionally, our research program offered rewards for reported recaptures which was advertised on the tags to increase the chance of reporting, especially by the TSR non-participants. While some recaptured sharks had tags that contained institutional information (e.g., NMFS Apex Predators Program), other tags did not have such information, or the reporter did not examine the tag closely enough to determine the tagging source. While pictures of the recaptured tag were requested, many reporters left the tag in the shark upon release without taking detailed pictures of the tag. As a result, we have recapture reports that cannot be verified due to an inability to determine the initial tagging source. This source of non-reporting prevents the acquisition of valuable data but can be addressed by tournament managers to minimize these occurrences in future tournaments. These tags along with other more sophisticated electronic tags show the need for a centralized tagging database, where “orphaned” tags can be paired with owners.

Finally, some recaptures were reported when a shark washed up on the beach dead, which comprised a relatively small percentage of all recaptures (9.8%). While the exact discard mortality is not known in the land-based shark fishery and needs further study, survivability is thought to be high. In fact, two sharks (a great hammerhead and a sandbar shark) have been recaptured multiple times during the study period, suggesting that some species of sharks may be more resilient than others [38–40], and/or some anglers were efficient and careful when handling and releasing sharks. Regardless, estimates of discard mortality in this increasingly popular land-based shark fishery are severely needed for stock assessments. While these estimates remain unavailable, outreach and engagement to provide anglers with the best handling and release practices are essential to increase survival and maintain a sustainable fishery.

## Conclusions

Citizen scientists have contributed valuable knowledge and data to wildlife and fisheries managers for decades. This study demonstrated that citizen scientists have the ability to tag, collect biological data, and identify sharks with little error, and that with the use of technology, such as digital cameras found in smartphones, identifications can be verified. As participants of larger tournaments, like the TSR, anglers collect substantial amounts of data throughout most of the year, allowing scientists and managers access to data on a geographic and temporal scale that could not be easily obtained otherwise. For anglers, this contribution can be done with little impact to the fishery as these anglers sampled fish caught using catch-and-release techniques during recreational fishing trips [37]. This is important for managers as these long-term datasets could potentially be incorporated into future assessments of sharks in the Gulf of Mexico. Future research should combine genetic barcoding to confirm species identification of unknown individuals and explore the possibility of cryptic or hybrid species misidentification, as well as determining the discard mortality in this land-based shark fishery.

## References

[1] Silverton J. A new dawn for citizen science. Trends Ecol Evol. 2009; 24:467–471. 10.1016/j.tree.2009.03.017 19586682

[2] Delaney DG, Sperling CD, Adams CS, Leung B. Marine invasive species: validation of citizen science and implications for national monitoring networks. Biol Invasions. 2008; 10: 117–128. 10.1007/s10530-007-9114-0

[3] Ajemian MJ, Jose PD, Froeschke JT, Wildhaber ML, Stunz GW. Was everything bigger in Texas? Characterization and trends of a land-based recreational shark fishery, Mar Coast Fish. 2016; 8: 553–566. 10.1080/19425120.2016.1227404

[4] Bray GS, Schramm HL. Evaluation of a statewide volunteer angler diary program for use as a fishery assessment tool. N Am J Fish Manag. 2001; 21: 606–615.

[5] Darwall WRT, Dulvy NK. An evaluation of the suitability of non-specialist volunteer researchers for coral reef fish surveys. Mafia Island, Tanzania—a case study. Biol Conserv. 1996; 78:223–231.

[6] Fore LS, Paulsen K, O’Laughlin K. Assessing the performance of volunteers in monitoring streams. Freshwater Biol. 2001; 46:109 10.1111/j.1365-2427.2001.00640.x

[7] Ohrel JR, Ronald L, Register KM. Volunteer estuary monitoring: a methods manual. 2nd ed Center for Marine Conservation, EPA; 2000.

[8] Wetz MS, Cira EK, Sterba-Boatwright B, Montagna PA, Palmer TA, Hayes KC. Exceptionally high organic nitrogen concentrations in a semi-arid South Texas estuary susceptible to brown tide blooms. Estuar Coast Shelf S. 2017; 188: 27–37. 10.1016/j.ecss.2017.02.001

[9] Butcher GS, Niven DK. Combining data from the Christmas bird count and the breeding bird survey to determine the continental status and trends of North America birds. National Audubon Society. 2007.

[10] Foster-Smith J, Evans SM. The value of marine ecological data collected by volunteers. Biol Conserv. 2003; 113: 199–213. 10.1016/S0006-3207(02)00373-7

[11] Stunz GW, Johnson MJ, Yoskowitz D, Robillard M, Wetz J. iSnapper: design, testing, and analysis of an iPhone‐based application as an electronic logbook in the for‐hire Gulf of Mexico Red Snapper fishery. National Oceanic and Atmospheric Administration Final Report NA10NMF4540111. 64 pp. 2014.

[12] Venturelli PA, Hyder K, Skov C. Angler apps as a source of recreational fisheries data: opportunities, challenges and proposed standards. Fish Fish. 2016; 18: 578–595. 10.1111/faf.12189

[13] Williams SM, Holmes BJ, Pepperell JG. The novel application of non-lethal citizen science tissue sampling in recreational fisheries. PLoS ONE. 2015; 10: 10.1371/journal.pone.0135743 26376487 PMC4572710

[14] Newman C, Buesching CD, Macdonald DW. Validating mammal monitoring methods and assessing the performance of volunteers in wildlife conservation-“Sed quis custodiet ipsos custodies?” Biol Conserv. 2003; 113: 189–197. 10.1016/S0006-3207(02)00374-9

[15] Saunders DA. Conservation research leads to a paradigm shift in farming practice: a case study from the Western Australian wheatbelt In: Lunney D, Dickman CR, Burgin S, editors. Community and research-based conservation: a clash of paradigms. Mosman: Royal Zoological Society of New South Wales 2002 pp. 54–63.

[16] Hunt E. New Zealand hunters apologise over accidental shooting of takahē. The Guardian. 21 8 2015 Available from: http://www.theguardian.com/environment/2015/aug/21/new-zealand-conservationists-apologise-over-accidental-shooting-of-endangered-takahe Cited 21 May 2019.

[17] Beerkircher L, Arocha F, Barse A. Effects of species misidentification on population assessment of overfished white marlin *Tetrapturus albidus* and roundscale spearfish *T*. *georgii*. Endanger. Species Res. 2009; 9, 81–90. 10.3354/esr00234

[18] Solow A, Smit W, Burgman M, Rout T, Wintle B, Roberts D. Uncertain sightings and the extinction of the ivory-billed woodpecker. Conserv. Biol. 2012; 26: 180–184. 10.1111/j.1523-1739.2011.01743.x 21967229

[19] Austen GE, Bindemann M, Griffiths RA, Roberts DL. Species identification by experts and non-experts: comparing images from field guides. Sci Rep. 2016; 6: 33634 10.1038/srep33634 27644140 PMC5028888

[20] Culverhouse PF, Williams R, Reguera, B, Herry V, González-Gil S. Do experts make mistakes? A comparison of human and machine identification of dinoflagellates. Mar. Ecol. Prog. Ser. 2003; 247: 17–25. 10.3354/meps247017

[21] Gibbon GEM, Bindemann M, Roberts DL. Factors affecting the identification of individual mountain bongo antelope. PeerJ. 2015; 3, e1303 10.7717/peerj.1303 26587336 PMC4647597

[22] Azzurro E, Broglio E, Maynou F, Bariche M. Citizen science detects the undetected: the case of *Abudefduf saxatilis* from the Mediterranean Sea. Manag Biol Invasion, 2013; 4(2): 167–170. 10.3391/mbi.2013.4.2.10

[23] RCoreTeam. 2014 R: a language and environment for statistical computing R foundation for statistical computing Vienna, Austria (http://www.R-project.org/).

[24] Kuhn M. Building predictive models in R using the caret package. J Stat Softw. 2008; 28: 1–26. 10.18637/jss.v028.i07 27774042 PMC5074077

[25] Williams CK, Engelhardt A, Cooper T, Mayer Z, Ziem A, Scrucca L, et al Package ‘caret’. 2019 Available from: https://cran.r-project.org/web/packages/caret/caret.pdf. Cited 21 May 2019.

[26] Aldrich CL. Shoreline management at Padre Island National Seashore: an investigation of angler relationships to the beach. Master’s thesis. Texas A&M University, College Station. 2009. Available from: https://core.ac.uk/download/pdf/4276208.pdf.

[27] Graefe AR, Ditton RB. Recreational shark fishing on the Texas Gulf coast: an exploratory study of behavior and attitudes. Marine Fisheries Review. 1976; 38:10–20.

[28] Gallagher AJ, Hammerschlag N, Danylchuk AJ, Cooke SJ. Shark recreational fishing: status, challenges, and research needs. Ambio. 2017; 46:385–398. 10.1007/s13280-016-0856-8 27995551 PMC5385669

[29] Jose PD. Population trends and migration patterns of the Texas nearshore shark assemblage. Master's thesis.Texas A&M University-Corpus Christi, Corpus Christi. 2014. Available from: https://www.sportfishcenter.org/sites/default/files/2018-07/jose_thesis_0.pdf.

[30] Gardiner MM, Allee LL, Brown PMJ, Losey JE, Roy HE, Smyth RR. Lessons from lady beetles: accuracy of monitoring data from US and UK citizen-science programs. Front Ecol Environ. 2012; 10:471–476. 10.1890/110185

[31] Fuccillo KK, Crimmins TM, de Rivera CA, Elder TS. Assessing accuracy in citizen science-based plant phenology monitoring. Int J Biometeorol. 2015; 59:914–926. 10.1007/s00484-014-0892-7 25179528

[32] Swanson A, Kosmala M, Lintott C, Packer C. A generalized approach for producing, quantifying, and validating citizen science data from wildlife images: citizen science data quality. Conserv Biol. 2016; 30:520–531. 10.1111/cobi.12695 27111678 PMC4999033

[33] Branstetter S. Problems associated with the identification and separation of the spinner shark, *Carcharhinus brevipinna*, and the blacktip shark, *Carcharhinus limbatus*. Copia. 1982; 2: 461–465.

[34] Lesueur CA. Description of a *Squalus*, of a very large size, which was taken on the coast of New Jersey. Journal of the Academy of Natural Sciences of Philadelphia. 1882; 2: 343–352.

[35] Parsons GR. Sharks, skates, and rays of the Gulf of Mexico. The University Press of Mississippi 2006 pp.165.

[36] Pine WE, Pollock KH, Hightower JE, Kwak TJ, Rice JA. A review of tagging methods for estimating fish population size and components of mortality. Fish. 2003; 28(10):10–23. 10.1577/1548-8446(2003)28[10:AROTMF]2.0.CO;2

[37] Guindon K, Neidig C, Tringali M, Gray S, King T, Gardinal C, et al An overview of the tarpon genetic recapture study in Florida—a citizen science success story. Environ Biol Fish. 2015; 98: 2239–2250. 10.1007/s10641-015-0440-2

[38] Gallagher AJ, Serafy JE, Cooke SJ, Hammerschlag N. Physiological stress response, reflex impairment, and survival of five sympatric shark species following experimental capture and release. Mar Ecol Prog Ser, 2014; 496: 207–218.

[39] Marshall H, Skomal G, Ross PG, Bernal D. At-vessel and post-release mortality of the dusky (*Carcharhinus obscurus*) and sandbar (*C*. *plumbeus*) sharks after longline capture. Fish Res. 2015; 172: 373–384.

[40] Morgan A, Burgess GH. At-vessel fishing mortality for six species of sharks caught in the northwest Atlantic and Gulf of Mexico. Gulf Caribb Res. 2007; 19: 123–129.

